# Increased HSD11β1 Expression in Human Leiomyomatous Uteri: Implication for Enhanced Glucocorticoid Signaling

**DOI:** 10.1210/clinem/dgaf255

**Published:** 2025-04-28

**Authors:** Carrie Malcom, Ozlem Guzeloglu-Kayisli, Burak Un, Erika New, Busra Cetinkaya-Un, Xiaofang Guo, Emad Mikhail, Anthony Imudia, Charles Lockwood, Umit Kayisli

**Affiliations:** Department of Obstetrics & Gynecology, University of South Florida, Morsani College of Medicine, Tampa, FL 33606, USA; Department of Obstetrics & Gynecology, University of South Florida, Morsani College of Medicine, Tampa, FL 33606, USA; Department of Obstetrics & Gynecology, University of South Florida, Morsani College of Medicine, Tampa, FL 33606, USA; Department of Obstetrics & Gynecology, University of South Florida, Morsani College of Medicine, Tampa, FL 33606, USA; Department of Obstetrics & Gynecology, University of South Florida, Morsani College of Medicine, Tampa, FL 33606, USA; Department of Obstetrics & Gynecology, University of South Florida, Morsani College of Medicine, Tampa, FL 33606, USA; Department of Obstetrics & Gynecology, University of South Florida, Morsani College of Medicine, Tampa, FL 33606, USA; Department of Obstetrics & Gynecology, University of South Florida, Morsani College of Medicine, Tampa, FL 33606, USA; Shady Grove Fertility Center of Tampa Bay, Wesley Chapel, FL 33544, USA; Department of Obstetrics & Gynecology, University of South Florida, Morsani College of Medicine, Tampa, FL 33606, USA; Department of Obstetrics & Gynecology, University of South Florida, Morsani College of Medicine, Tampa, FL 33606, USA

**Keywords:** FKBP51, HSD11β1, leiomyoma, glucocorticoid signaling, gene expression

## Abstract

**Context:**

FK506-binding-protein-51 (FKBP51) is a glucocorticoid-induced co-chaperone protein previously shown to bind glucocorticoid receptor (GR), inhibiting its transcriptional activity. We previously found increased FKBP51 levels in uterine leiomyoma vs paired myometrium.

**Objective:**

To test the hypothesis that elevated FKBP51 levels contribute to leiomyoma pathogenesis by altering GR signaling.

**Design:**

RNA-sequencing was performed in leiomyoma cell cultures transfected with scramble or *FKBP5*-siRNA for 48 hours, then treated with vehicle or dexamethasone (DEX) for 24 hours. Differentially expressed genes, including *HSD11B1, CNN1,* and *LAMA2* were analyzed by quantitative polymerase chain reaction. Hydroxysteroid 11-β dehydrogenase 1 (HSD11β1) expression was analyzed in leiomyoma, leiomyoma-adjacent paired myometrium, myometrium from patients without leiomyoma, and human endometrial stromal cells by quantitative polymerase chain reaction and immunohistochemistry.

**Setting:**

University research institution.

**Patients:**

Women with or without uterine leiomyoma.

**Main Outcome Measures:**

*HSD11B1* mRNA and protein levels in leiomyoma, paired myometrium, and normal myometrium.

**Results:**

HSD11β1 expression was higher in paired myometrial and leiomyoma tissues vs normal myometrium (*P* < .02). DEX treatment increased *HSD11B1* transcription in normal myometrial and human endometrial stromal cell cultures, but to a significantly greater extent in leiomyoma (*P* < .001). However, *FKBP5*-silencing blunted this DEX-induced *HSD11B1* upregulation. DEX-treatment reduced *LAMA2* and increased *CNN1* levels (coding for extracellular matrix and smooth muscle proteins, respectively) in *FKBP5*-silenced vs scramble siRNA-transfected leiomyoma cultures.

**Conclusion:**

FKBP51 not only inhibits but can augment GR-mediated transcription. Importantly, FKBP51-GR interactions increase *HSD11B1* levels in leiomyoma cells, generating a pathological FKBP51-GR-HSD11β1 circle, altering transcription of downstream extracellular matrix and smooth muscle genes to induce a myofibroblast phenotype, thereby possibly contributing to leiomyoma pathogenesis.

Uterine leiomyomas are a common pathology seen in up to 70% women by menopause, and cause pelvic pain, abnormal uterine bleeding, and infertility (1, 2). It has been previously shown that estrogen and progesterone signaling induce proliferation of leiomyoma cells (3, 4). Current treatments to mitigate leiomyoma growth and associated bleeding primarily involve sex steroids, including combined estrogen-progesterone oral contraceptives, progestins, and progesterone receptor antagonists (2, 5). Even though newer agents like GnRH agonists and antagonists effectively control symptoms (6), they cannot be used long-term and must be discontinued in patients attempting to conceive. Thus, it is crucial to develop therapeutic options that do not interfere with reproduction. Achieving this goal requires a deeper understanding of the mechanisms underlying leiomyoma pathogenesis.

FK506-binding-protein-51 (FKBP51) is a co-chaperone protein encoded by the *FKBP5* gene, which contains glucocorticoid and progesterone response elements, though *FKBP5* is predominantly induced by glucocorticoids (7). Glucocorticoid-induced FKBP51 binds to both the glucocorticoid receptor (GR) and the progesterone receptor (PR), inhibiting GR- and PR-mediated transcriptional activity by impeding ligand binding, a phenomenon that has been unequivocally reported in numerous studies published (8-11). Previously, we demonstrated that glucocorticoids increase FKBP51 levels in both human decidual and endometrial stromal cells (12, 13). More recently, we found elevated expression of FKBP51 in leiomyomas vs paired myometrial tissue (14).

The role of glucocorticoid signaling in the pathogenesis of leiomyoma has been investigated. Whirledge et al found that dexamethasone (DEX) increased *FKBP5* levels, reduced the number of cells in S-phase, and inhibited the expression of the estrogen receptor along with genes regulating cell replication in immortalized human uterine leiomyoma cells (4). Moreover, mifepristone, which is a competitive antagonist at both PR and GR with more than 3 times greater binding affinity for GR than DEX (15), inhibits extracellular matrix (ECM) formation in uterine leiomyoma (2). Together, these findings, along with our previous results, suggested that elevated glucocorticoid and/or progesterone signaling contribute to leiomyoma pathogenesis by increasing FKBP51 levels and promoting ECM formation.

In the current study, we sought to clarify the role of FKBP51 on glucocorticoid signaling in leiomyoma. We first investigated FKBP51's effect on global GR-mediated transcriptional activity in primary leiomyoma cell cultures. This analysis revealed that: (1) DEX induces *HSD11B1*, which converts cortisone to its active form cortisol (16); and (2) *FKBP5* silencing diminished DEX-mediated induction *of HSD11B1*. This finding contradicts prior studies reporting that FKBP51 consistently inhibits GR transcriptional activity (9, 17).

Thus, to better understand the dysregulation of FKBP51-GR signaling in the pathogenesis of leiomyoma, we focused on downstream regulation of hydroxysteroid 11-β dehydrogenase 1 (HSD11β1) in normal myometrium vs leiomyoma tissues and found increased HSD11β1 expression in leiomyoma samples compared to normal myometrium. These findings led to the proposal of a novel hypothesis that elevated glucocorticoid signaling in leiomyomatous uteri creates a local positive feedback loop to enhance FKBP51-GR interactions thereby upregulating HSD11β1, leading to increased cortisol production. Cortisol, in turn, further activates GR signaling and promotes additional FKBP51 expression. In vitro studies confirmed that this local positive feedback loop induces a switch from a smooth muscle to myofibroblast phenotype, a well-known characteristic of leiomyoma cells.

## Methods and Materials

### Collection of Human Specimens

All uterine samples were collected following patient consent and approved by the institutional review board of the University of South Florida (#Pro00021684). Paired uterine leiomyoma and adjacent normal myometrial tissues were collected >1 cm apart from each other as described in detail by New et al (14). Normal myometrial specimens were collected from premenopausal patients without leiomyoma who were undergoing hysterectomy for endometriosis, pelvic pain, benign adnexal mass, or cervical dysplasia, and had not received hormonal medications within 3 months before surgery. Endometrial samples were collected from these patients via a Pipelle suction curette before hysterectomy or from scrapings of the endometrial cavity after hysterectomy.

### Cell Cultures

Frozen aliquots of previously isolated, cultured, and characterized primary leiomyoma cells, as described by New et al (14). were grown in basal media including phenol-red free DMEM/F12 (Thermo Fisher Scientific, Waltham, MA) supplemented with 10% fetal bovine serum (GeminiBio, West Sacramento, CA) and 1% antibiotic and antimycotics (Thermo Fisher Scientific) until reaching confluency.

Normal myometrial tissue samples were collected in Hanks’ balanced salt solution (Thermo Fisher Scientific) within 1 hour of surgery. The myometrial tissues were minced and digested in a buffer containing 0.2 mg/mL deoxyribonuclease I DNase I (Roche Diagnostics, Indianapolis, IN) and 5 mg/mL collagenase type II (5 mg/mL; Thermo Fisher Scientific) for 45 minutes at 37 °C with gentle agitation. The digested tissue pieces were then plated into 10-cm tissue culture dishes and cultured in basal media supplemented with 10 ng/mL epidermal growth factor (R&D Systems, Minneapolis, MN) until the primary outgrowing cells reached approximately 70% confluency. For subculturing, the growing cells were trypsinized using 0.25% trypsin and 0.05% EDTA (Thermo Fisher Scientific). The smooth muscle-like integrity of cultured myometrial cells was previously confirmed as detailed by New et al (14).

Endometrial tissue samples were collected in Hanks’ balanced salt solution within 1 hour of surgery to isolate human endometrial stromal cells (HESCs), which were isolated and cultured as described previously by Pavlovic et al (18). Briefly, minced endometrial tissues were digested in DMEM/F12 containing collagenase B (1 mg/mL, 15 U/mg (Roche), deoxyribonuclease I (0.1 mg/mL, 1500 U/mg; Roche), penicillin (200 U/mL), and streptomycin (200 mg/mL) for 30 minutes at 37 °C with gentle agitation. The dispersed endometrial cells were separated by filtration through a 70-µm diameter cell strainer (Sigma-Aldrich, St. Louis, MO) and cultured in basal media until grown to confluence.

### Silencing of FKBP5 by siRNA and Culture Treatments

Leiomyoma (n = 4), normal myometrial (n = 4), and endometrial stromal cell (n = 4) cultures seeded at 1.5 × 10^5^ cells/well were incubated in basal media for 24 hours in a 6-well plate. The next day, cells were transfected with 20 nM of nonspecific control (scramble) siRNA (Invitrogen, Thermo Fisher Scientific) or *FKBP5*-specific siRNA (Santa Cruz Biotechnology, Inc., Dallas, TX) using lipofectamine RNAiMax transfection reagent (Thermo Fisher Scientific) in Opti-MEM I serum reduced medium (Thermo Fisher Scientific) as described by Sprague et al (19). Forty-eight hours after transfection, cells were treated with either vehicle medium (DMEM/F12 with 2% fetal bovine serum) or 10^−7^ M DEX (Sigma-Aldrich) for 24 hours then washed with ice-cold PBS and stored at −80 °C for RNA isolation. Transfection efficiency for silencing of *FKBP5* mRNA and protein was confirmed by real-time quantitative polymerase chain reaction (qPCR) and immunoblotting as described by New et al (14).

### RNA Isolation and Reverse Transcription

Total RNAs were isolated from: (1) leiomyoma and paired myometrial tissues from the proliferative (n = 9) and secretory (n = 8) phases; (2) normal myometrial tissues from the proliferative (n = 5) and secretory (n = 11) phases; and (3) cultured leiomyoma (n = 4), normal myometrial (n = 4), and human endometrial stromal cells (n = 4) that were previously transfected with control or *FKBP5* siRNA ± DEX, using the miRNeasy Mini Kit (Qiagen Inc, Germantown, MD). Reverse transcription was performed using the Qiagen Omniscript RT kit (Qiagen) according to the manufacturer's instructions.

### RNA Sequencing and Analysis

RNA samples from control or *FKBP5* siRNA-transfected ± 10^−7^ M DEX-treated primary leiomyoma cultures (n = 4) were equally pooled and processed for whole-genome RNA sequencing by Novogene, Inc. (Sacramento, CA) using the NovaSeq 6000. RNA samples with an RNA Integrity Number value > 8 were used for RNA-sequencing (RNA-seq) analysis. After normalization, genes with a fold change of >1.5 and a *P* value <.05 were considered differentially expressed in DEX-treated control or *FKBP5*-silenced vs vehicle-treated control or *FKBP5*-silenced cells. RNA-seq data sets can be found in the NCBI GEO repository, accession number GSE292403 (20). Upstream regulator analysis in differentially expressed genes and creation of heat map and volcano plots were performed using Ingenuity Pathway Analysis (IPA) software (Qiagen). qPCR was performed in these cultures (n = 4) to confirm differentially expressed genes.

### Real Time Quantitative PCR

Using gene-specific TaqMan gene expression assays (Applied Biosystem, Foster City, CA), qPCR was performed as described by Guzeloglu-Kayisli et al (12). The full name and Taq-Man Probe Assay IDs for each target gene include: FK506-binding protein 5 (*FKBP5*)- Hs01561006_m1; Hydroxysteroid 11-β dehydrogenase 1 (*HSD11B1)*- Hs01547870_m1; IL-1 receptor type 1 (*IL1R1*)- Hs00991002_m1; TSC22 domain family member 3 (*TSC22D3*)- Hs00608272_m1; alcohol dehydrogenase 1B, β polypeptide (*ADH1B*)- Hs00605175_m1; Gremlin 1, DAN family BMP antagonist (*GREM1*)- Hs00171951_m1; IGF-binding protein 5 (*IGFBP5*)- Hs00181213_m1; adhesion molecule with Ig-like domain 2 (*AMIGO2*)- Hs05001325_s1; laminin subunit alpha 2 (*LAMA2*)- Hs00166308_m1; fibronectin 1 (*FN1*)- Hs00365052_m1; calponin 1 (*CNN1*)- Hs00959434_m1; actin β (*ACTB*)- Hs99999903_m1; and glyceraldehyde-3-phosphate dehydrogenase (*GAPDH*)- Hs99999905_m1. All samples were run in duplicate, with *ACTB* and *GAPDH* used as endogenous control to normalize the target genes. Relative fold change was calculated using the 2^−ΔΔCT^ method.

### Immunohistochemistry

Immunostaining was performed to detect endogenous HSD11β1 levels in leiomyoma (n = 6), paired myometrial (n = 6), and normal myometrial tissues (n = 6). Leiomyoma, paired myometrial, and normal myometrial tissue samples consisted of both mid-proliferative (n = 3) and mid-secretory (n = 3) phases. Five-micrometer serial sections of 4% paraformaldehyde fixed and paraffin-embedded tissue samples were deparaffinized and rehydrated, then incubated in 3% hydrogen peroxide for blockage of endogenous peroxidase activity as described by New et al (14). Antigen retrieval was then performed by boiling in Tris-EDTA buffer solution (10 mM Tris base, 1 mM EDTA; pH 9.0) for 20 minutes. After washing in Tris-buffered saline (TBS), slides were incubated with 5% normal goat serum (Vector Labs, Burlingame, CA) for 30 minutes at room temperature, then incubated overnight at 4 °C in rabbit polyclonal antibody against HSD11β1 (1:100, Atlas Antibodies Cat# HPA047729, RRID:AB_2680135) in 2.5% goat serum. As a negative control, sections were incubated in an equivalent concentration of nonspecific rabbit IgG (Cell Signaling, Danvers, MA). Sections were washed in TBS with 1% Tween 20, then incubated with biotinylated goat anti-rabbit secondary IgG (1:400; Vector Labs) for 30 minutes at room temperature. Antigen-antibody complexes were visualized following incubation with streptavidin-biotin-peroxidase complex (Elite ABC kit; Vector Labs) for 30 minutes and then chromogen 3,3-diaminobenzidine (Vector Labs, Newark, CA) for 5 minutes at room temperature. Nuclei were stained with hematoxylin. The slides were then covered using an aqueous-based mounting medium.

### Statistical Analysis

Data were analyzed with SigmaStat version 11.0 software (Systat Software, Inc, San Jose, CA) using pair-wise multiple comparisons by 1-way ANOVA followed by the post hoc Tukey test if parametrically distributed or by Student-Newman-Keuls if nonparametrically distributed. The *t*-test or Mann-Whitney-Wilcoxon rank-sum test were used for comparison of 2 groups for parametric or nonparametric distribution, respectively. Statistical significance was defined as *P* < .05.

## Results

### *FKBP5* Knockdown Blunts GR-mediated Transcriptional Activity in Leiomyoma Cells

**Figure 1.**
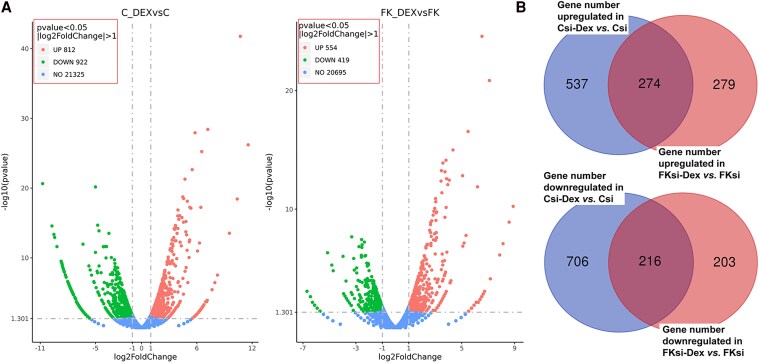
*FKBP5*-silencing reduces dexamethasone-induced differentially regulated genes in leiomyoma cells. (A) Volcano plots of RNA-sequencing data (n = 4 each group) comparing control leiomyoma cells (C) or cells transfected with *FKBP5* siRNA (FK) with dexamethasone (DEX)-treated cells (C-DEX vs C or FKD-DEX vs FKD) show differentially up- or down- regulated genes expressed as log2 fold change. (B) Venn diagrams display the specific number of genes that are upregulated and downregulated in DEX-treated control or *FKBP5* knockdown cells vs control or *FKBP5* knockdown cells, respectively; and which genes are uniquely up- or down- regulated or common between the 2 groups. RNA sequencing raw data available in the NCBI GEO repository, accession number GSE292403 (20).

To understand how FKBP51 contributes to the pathogenesis of leiomyoma, specifically its impact on global GR-mediated transcriptional activity, we conducted studies in primary leiomyoma cultures. Primary cultured leiomyoma cells were transfected with scramble (control) siRNA vs *FKBP5* siRNA for 48 hours, then treated with either vehicle or 10^−7^ M DEX for 24 hours, followed by analysis of RNA by whole-genome RNA-seq. To assess transfection efficiency, *FKBP5* mRNA levels were measured and found to be reduced by 13-fold in *FKBP5* siRNA-transfected leiomyoma cell cultures compared to those transfected with scramble siRNA (.08 ± 0.04 vs 1.04 ± 0.14; *P* < .001). FKBP51 protein levels were also analyzed via immunoblotting, showing reduced levels in *FKBP5* siRNA-transfected leiomyoma cells vs scramble siRNA-transfected cells (14). Following RNA-seq, gene expression was compared between control siRNA-transfected cells treated with DEX vs vehicle (C-DEX vs C) as well as between *FKBP5* siRNA-transfected cells treated with DEX vs vehicle (FKD-DEX vs FKD). After normalization as described previously (21, 22), expression levels of genes with |log2FoldChange| > 1 were considered differentially expressed genes (DEGs) and displayed in Volcano plots (Fig. 1A). Contrary to the expectation that reducing FKBP51 levels would enhance glucocorticoid-induced, GR-mediated transcriptional activity (8-11), in *FKBP5*-silenced compared to control siRNA-transfected cells, DEX treatment significantly reduced the number of upregulated (812 vs 554 DEGs) genes, as well as downregulated genes (922 vs 419 DEGs) (Fig. 1A). Venn diagram analysis revealed that: (1) DEX treatment upregulated 537 genes in control cells, whereas only 279 genes were upregulated in *FKBP5*-silenced cells, with 274 genes common to both groups; and (2) in DEX-treated control cells, 706 genes were downregulated, compared to only 203 genes in DEX-treated *FKBP5*-silenced cells, with 216 genes common to both groups (Fig. 1B; full list of DEGs for each Volcano plot can be found in the NCBI GEO repository, accession number GSE292403 (20)).

**Figure 2.**
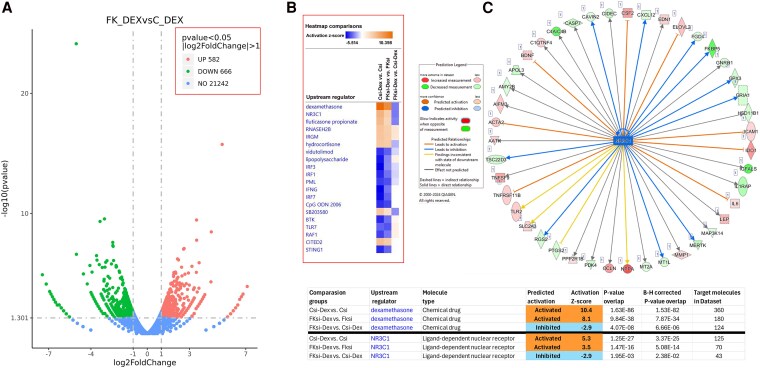
*FKBP5* knockdown in leiomyoma cells treated with dexamethasone predicts inhibition of glucocorticoid signaling. (A) Volcano plot of RNA-sequencing data (n = 4 each group) directly compares differential gene expression in dexamethasone (DEX)-treated *FKBP5*-silenced (FKD_DEX) vs control (C_DEX) leiomyoma cells. (B) Heat map depicts the activation *Z*-score for RNA-sequencing data, comparing DEX-treated control (Csi-Dex) vs vehicle-treated control leiomyoma cells (Csi), DEX-treated *FKBP5*-silenced (FKsi-Dex) vs vehicle-treated *FKBP5*-silenced leiomyoma cells (FKsi), and DEX-treated *FKBP5*-silenced vs DEX-treated control cells (FKsi-Dex vs Csi-Dex). Silencing of *FKBP5* results in the inhibition of dexamethasone and glucocorticoid receptor (*NR3C1*) downstream signaling. (C) Ingenuity Pathway-Upstream Regulator Analysis indicates that the upstream target *NR3C1* is predicted to be inhibited in DEX-treated *FKBP5*-silenced vs DEX-treated control (FKsi-Dex vs Csi-Dex) leiomyoma cells. RNA sequencing raw data available in the NCBI GEO repository, accession number GSE292403 (20).

Given these unexpected findings in comparisons of C-DEX vs C and FKD-DEX vs FKD, we next compared DEX-treated *FKBP5*-silenced cells (FKD-DEX) with DEX-treated control cells (C-DEX). This analysis identified a total of 1248 DEGs, with 582 genes upregulated and 666 genes downregulated (Fig. 2A; see the full list of DEGs in the NCBI GEO repository, accession number GSE292403 (20)).

Using IPA software (Qiagen), we performed heatmap comparison (Fig. 2B) and assessed the activation and inhibition status of DEX- and GR (*NR3C1*)-mediated pathways as upstream regulators of DEGs. This assessment was based on the *Z*-score, with a positive value >2 indicating activation and a negative value < −2 indicating inhibition, in the comparisons of C-DEX vs C, FKD-DEX vs FKD, and FKD-DEX vs C-DEX (Fig. 2B). These comparisons revealed that compared to control siRNA-transfected cells, *FKBP5* knockdown reduced the *Z*-score of DEX from 10.4 to 8.1 in regulating its downstream genes, and decreased *Z*-score of NR3C1 from 5.3 to 3.5 for its downstream genes (Fig. 2B). These reduced *Z*-scores suggest that *FKBP5* knockdown inhibits DEX and/or NR3C1-mediated transcriptional activity. This observation is further supported by the finding that compared to control DEX-treated cultures, *FKBP5*-silenced DEX-treated cultures, exhibited a *Z*-score of −2.9 for both DEX and NR3C1, indicating inhibition of their activity as upstream regulator of DEGs (Fig. 2B). According to the IPA analysis, the inhibitions of DEX and NR3C1 activity in the FKD-DEX vs C-DEX group were linked to 124 and 43 DEGs, respectively (Fig. 2B and C). Overall, these findings contrast markedly with previous studies that reported a consistent inhibitory effect of FKBP51 on GR transcriptional activity (8-11).

**Figure 3.**
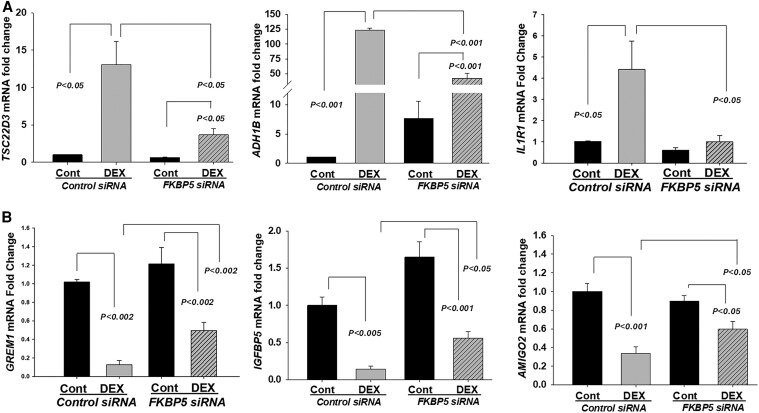
Silencing of *FKBP5* blunts dexamethasone (DEX)-regulated expression of glucocorticoid response element-containing genes in primary leiomyoma cell cultures. qPCR analysis of mRNA levels for DEX-upregulated genes *TSC22D*3*, ADH1B, IL1R1* (A), and DEX-downregulated genes *GREM1, IGFBP1, AMIGO2* (B) in cultured leiomyoma cells transfected with either control or *FKBP5* siRNA for 48 hours and then treated with either vehicle (Cont) or DEX for 24 hours. Bars represent mean ± standard error of the mean, n = 4.

To confirm these unexpected global RNA-seq findings, we conducted qPCR analysis on DEGs known to contain a GR response element (23, 24), including DEX-upregulated genes *TSC22D3, ADH1B*, and *IL1R1* (Fig. 3A) and DEX-downregulated genes *GREM1, IGFBP5*, and *AMIGO2* (Fig. 3B). These analyses confirmed the leiomyoma-associated dysfunctional regulation of GR transcriptional activity following *FKBP5* silencing, affecting both DEX-induced and DEX-suppressed genes (Fig. 3A and B).

### FKBP51-dependent Upregulation of *HSD11B1* Expression by DEX in Leiomyoma Cells

**Figure 4.**
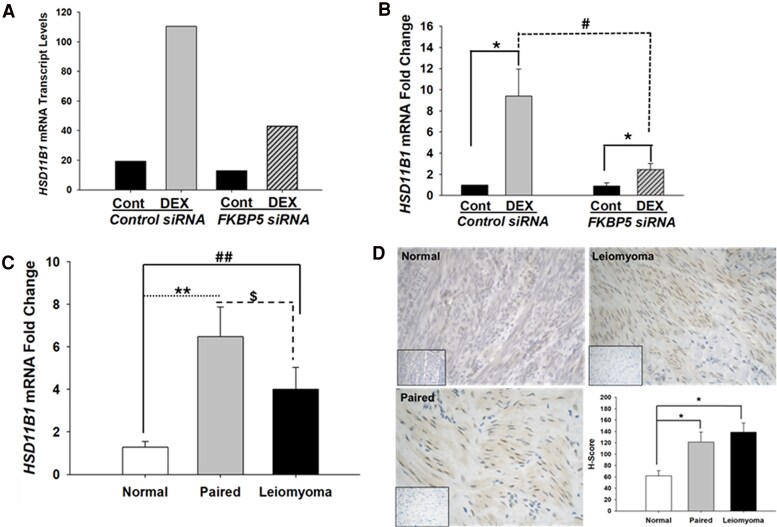
FKBP51 promotes increased *HSD11B1* expression, with baseline *HSD11B1* levels elevated in leiomyomatous uteri. *HSD11B1* levels in leiomyoma cells (n = 4) (A) by RNA-sequencing representing fragments per kilobase of transcript per million mapped reads (FPKM) and (B) by qPCR. Dexamethasone (DEX) treatment increased *HSD11B1* mRNA levels in control siRNA-transfected cells vs vehicle treatment (Cont). However, this DEX-induced upregulation was blunted in *FKBP5-*silenced cells. (C) In situ *HSD11B1* mRNA levels are increased in paired leiomyoma tissue samples (n = 17 paired, n = 17 leiomyoma) relative to normal myometrium samples (n = 16). (D) HSD11β1 immunostaining (brown) in normal myometrium (n = 6), paired (n = 6) and leiomyoma (n = 6) tissue samples, with decreased expression seen in normal tissues as quantified by H score. Inset represents negative control in each tissue sample. Bars represent mean ± standard error of the mean. *P < .05, ^#^*P* < .001, **P = .002, ^##^*P = .01*, ^$^*P = .04.* RNA sequencing raw data available in the NCBI GEO repository, accession number GSE292403 (20).

Among the DEGs, *HSD11B1* was a key downstream target that showed decreased expression following *FKBP5* silencing. The whole-genome sequencing results indicated a 5.7-fold increase in *HSD11B1* expression in leiomyoma cells treated with DEX compared to vehicle controls, whereas this increase was only 3.3-fold in *FKBP5*-knockdown cells (Fig. 4A; RNA-seq raw data available in the NCBI GEO repository, accession number GSE292403 (20)). Confirmational analysis by qPCR revealed a significant enhancement of *HSD11B1* levels in DEX-treated control cells, showing a 9.4-fold increase compared to control (*P* < .05; Fig. 4B). However, in *FKBP5* siRNA-transfected leiomyoma cells, DEX treatment produced a lesser (2.5-fold) increase in *HSD11B1* levels compared to control (*P* < .05) (Fig. 4B). This increase was significantly lower than that detected in DEX-treated control siRNA transfected cells (*P* < .001; Fig. 4B). Collectively, these findings suggest that FKBP51 plays a role in facilitating GR-mediated signaling in leiomyoma cells through increases in HSD11β1.

### In Situ Levels of HSD11β1 mRNA and Protein Expression are Increased in Leiomyomatous Uteri

We next analyzed HSD11β1 mRNA and protein levels in paired myometrial and leiomyoma tissue samples from patients with leiomyomas, as well as in normal myometrial samples from patients without leiomyomas. Analysis of qPCR results revealed that paired myometrium from uteri with leiomyomas exhibited a 6.5-fold increase in expression relative to normal myometrium (*P* = .002), while leiomyoma samples showed a 4-fold increase (*P* = .01) (Fig. 4C). The difference between paired myometrium vs leiomyoma tissues was also significant (*P* = .04). When patient samples were categorized by menstrual cycle phase, those in the proliferative phase exhibited higher *HSD11B1* expression than those in the secretory phase across all 3 groups, although this difference did not attain a statistical significance (this study was underpowered for such a comparison). Further analysis using immunohistochemistry in paired myometrial and leiomyoma tissue sections, as well as normal myometrium sections, confirmed that HSD11β1 protein levels mirrored the mRNA findings, with paired myometrium and leiomyoma tissues having significantly higher expression than normal myometrium (*P* < .05; Fig. 4D).

### DEX-induced HSD11B1 Expression by FKBP51-GR Signaling Is More Pronounced in Leiomyoma Cells Than Normal Myometrial and Human Endometrial Stromal Cells

**Figure 5.**
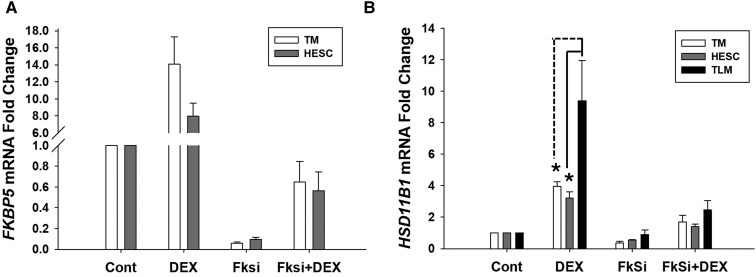
Dexamethasone (DEX) treatment increases *HSD11B1* expression in normal myometrial and human endometrial stromal cell cultures but to a lesser extent than in leiomyoma cells, in a FKBP51-dependent manner. (A) *FKBP5* knockdown efficiency in normal myometrial cell (TM; n = 4) and human endometrial stromal cell (HESC; n = 4) cultures was confirmed by qPCR, with significantly decreased *FKBP5* mRNA levels seen in *FKBP5* siRNA-transfected cells (FkSi) vs control siRNA-transfected cells (Cont). DEX treatment increased *FKBP5* mRNA levels in TM and HESC cell lines. This DEX-induced increase in *FKBP5* levels was less pronounced in *FKBP5*-silenced cells (FkSi + DEX) for both cell lines. (B) DEX treatment increased *HSD11B1* mRNA levels in control siRNA-transfected TM (n = 4), HESC (n = 4), and leiomyoma (TLM; n = 4) cells (DEX) vs vehicle treatment (Cont). This increase was blunted following *FKBP5* knockdown (FkSi + DEX vs FkSi; fold change relative to Cont). Bars represent mean ± standard error of the mean. *P < .05.

To determine whether the intracellular relationship between FKBP51 and HSD11β1 is specific to leiomyoma cells, cell culture experiments evaluating *HSD11B1* mRNA expression in leiomyoma cells transfected with control vs *FKBP5* siRNA and treated with DEX were repeated in cultured normal myometrial cells and HESCs obtained from uteri without leiomyoma. The efficiency of *FKBP5* knockdown in these cell cultures was confirmed by qPCR. In normal myometrial cultures, *FKBP5* siRNA-transfected cells showed a 17-fold reduction in *FKBP5* mRNA levels (0.058 ± 0.01 vs 1.0 ± 0, *P* < .001), whereas this reduction was 10.5-fold in HESCs (0.095 ± 0.02 vs 1.0 ± 0, *P* < .001) compared to scramble siRNA-transfected cell lines (Fig. 5A). Similarly, DEX increased *HSD11B1* mRNA levels by 4-fold in normal myometrial cells and by 3.2-fold in HESCs; however, these increases were significantly lower than the 9.4-fold upregulation observed in leiomyoma cells (*P* < .05; Fig. 5B). Interestingly, following *FKBP5* knockdown, *HSD11B1* levels were upregulated by DEX in these 3 cells, but the increases were substantially lower than those seen in control siRNA-transfected cells: 1.7-fold in normal myometrial cells (*P* < .05), 1.4-fold in HESCs (*P* < .05), and 2.5-fold in leiomyoma cells (*P* < .05). However, these increases in *HSD11B1* levels were not significantly different between the 3 cell types (*P* = .248; Fig. 5B). These findings suggest that DEX-induced GR signaling increases *HSD11B1* expression in a FKBP51-dependent manner across different types of uterine cells, with leiomyoma cells exhibiting a much greater response in the presence of FKBP51.

### Enhanced FKBP51-glucocorticoid Signaling Induces Leiomyoma Cells Toward a Myofibroblast Phenotype

**Figure 6.**
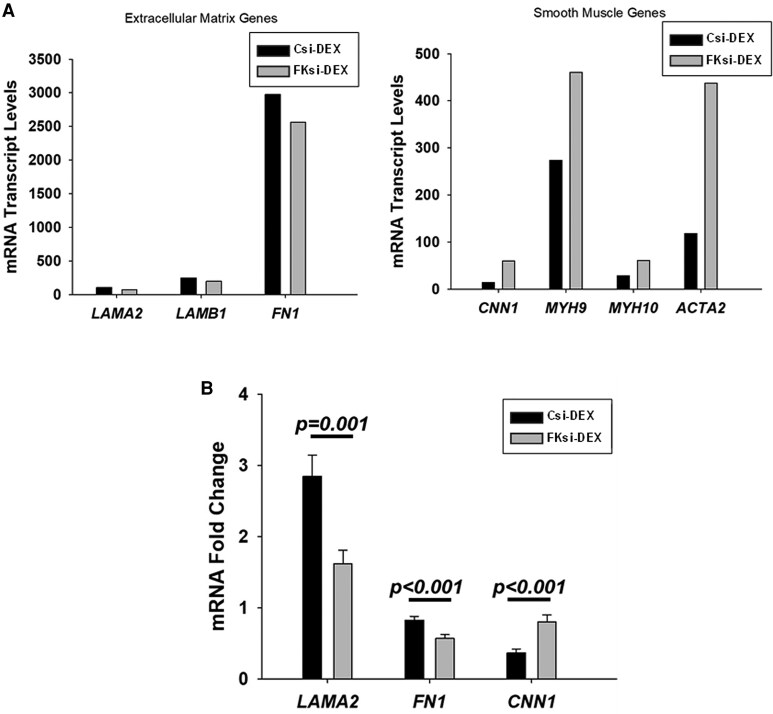
FKBP51 and dexamethasone induce a switch in leiomyoma cells from a smooth muscle to myofibroblast phenotype. (A) RNA-sequencing analysis of cultured leiomyoma cells (n = 4) shows lower expression levels of extracellular matrix genes including *LAMA2*, *LAMB1*, and *FN1*, and higher expression levels of smooth muscle expressed genes *CNN1*, *MYH9*, *MYH10,* and *ACTA2* following dexamethasone (DEX) treatment in *FKBP5* siRNA (FKsi) vs control siRNA (Csi) transfected cells. (B) Confirmation of significantly lower expression levels of *LAMA2* and *FN1*, and higher expression levels of *CNN1* in FKsi-Dex vs Csi-Dex by qPCR analysis. Bars represent mean ± standard error of the mean; n = 4. RNA sequencing raw data available in the NCBI GEO repository, accession number GSE292403 (20).

To elucidate how elevated FKBP51-GR signaling contributes to the pathogenesis of leiomyomas, we compared the expression of genes encoding ECM proteins (*LAMA2, LAMB1,* and *FN1*) and genes associated with smooth muscle cell proteins *(CNN1, MYH9, MYH10, ACTA2*) in cultured leiomyoma cells transfected with control vs *FKBP5* siRNA and treated with DEX. The RNA sequencing data revealed that in FKD-DEX vs C-DEX, transcription levels of ECM genes were reduced, whereas genes encoding proteins expressed by smooth muscle cells were increased (Fig. 6A; raw data available in the NCBI GEO repository, accession number GSE292403 (20)). To confirm these findings, expression levels of *LAMA2, FN1, and CNN1* were analyzed by qPCR. The results indicated that DEX treatment resulted in significantly lower levels of *LAMA2* (*P* = .001) and *FN1* (*P* = .001) in *FKBP5*-silenced cells vs control cells. Conversely, *CNN1* levels were significantly higher in DEX-treated *FKBP5*-silenced cells vs DEX-treated control cells (*P* < .001; Fig. 6B). These findings suggest that increased FKBP51-GR signaling induces the switch from a smooth muscle to a myofibroblast phenotype in leiomyoma cells.

## Discussion

**Figure 7.**
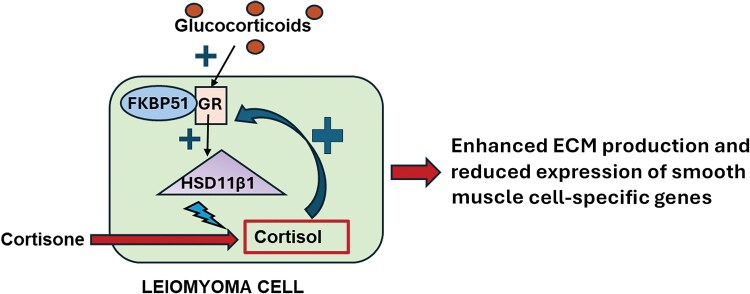
Schematic illustration of the potential mechanism by which enhanced FKBP51-GR signaling contributes to the pathogenesis of leiomyoma. Elevated levels of FKBP51 augment glucocorticoid-induced GR-mediated upregulation of HSD11β1, which converts inactive cortisone to active cortisol, leading to sustained intracellular glucocorticoid signaling in leiomyoma cells. This FKBP51-GR-HSD11β1 relationship generates an intracellular pathological circle, leading to increased expression of extracellular matrix (ECM) genes and decreased levels of genes expressed by smooth muscle cells, supporting a switch from a smooth muscle to myofibroblast phenotype, thereby contributing to the pathogenesis of leiomyoma.

The current study sought to understand how FKBP51-GR signaling interactions contribute to the pathogenesis of leiomyoma. Analysis of whole-genome RNA-seq in primary leiomyoma cell cultures showed that FKBP51 amplifies GR-mediated transcriptional effects, both up- and down-regulating numerous genes. These findings contrast with existing literature, which suggests that FKBP51 universally inhibits GR-mediated transcriptional activity (9, 17, 25, 26). Our RNA sequencing analysis identified *HSD11B1* as a key mediator of this enhanced FKBP51-GR signaling. In vitro and in situ results confirmed increased HSD11β1 levels in leiomyomatous uteri compared to normal myometrium. Mechanistically, we demonstrated that increased FKBP51-GR signaling in leiomyoma cells induces a switch from a smooth muscle to myofibroblast phenotype. This is consistent with previous studies that have documented increased deposition of ECM proteins in uterine leiomyoma (2, 14). Our findings suggest that elevated GR signaling in leiomyomatous uteri creates a local positive pathological feedback loop wherein increased FKBP51 levels result in upregulation of HSD11β1, thereby generating active cortisol. This process sustains GR activation and further promotes FKBP51 while also inducing expression of ECM genes and inhibiting genes encoding smooth muscle proteins (Fig. 7).

Many prior studies showing that FKBP51 inhibits GR signaling were performed in neuronal tissues, linking a role for FKBP51 in the pathogenesis of Alzheimer disease (27), posttraumatic stress disorder, and depression (17, 26). Studies from our laboratory established a role for FKBP51 inhibition of PR and GR signaling in decidual and endometrial stromal cells (7, 12, 13). Our current findings of increased FKBP51-GR signaling through an upregulation of HSD11β1 in leiomyoma cells suggest that this phenomenon may be specific to leiomyoma cells. Prior studies have noted differential gene expression in different cell types following DEX treatment (lung carcinoma A549 vs osteosarcoma U2OS cells lines) (28). However, we observed the same phenomenon in normal uterine myometrial and endometrial stromal cells, though to a lesser degree, suggesting this effect may be specific to uterine tissue. Further studies will need to be performed in other cell types including neuronal cells to see if this FKBP51-GR induction of HSD11β1 occurs in these tissue types as well, potentially offering a new therapeutic avenue for these psychiatric conditions.

HSD11β1, however, is not the only enzyme regulating intracellular glucocorticoid activity. HSD11β2 also plays a role, as it has opposing activity, converting active cortisol to inactive cortisone. The amounts of these 2 enzymes are tissue- and cell type-specific (29). Thus, the balance between the 2 will influence the extent of GR signaling. In our leiomyoma cell culture RNA-seq data, *HSD11B2* transcript levels were extremely low, representing less than 0.1 fragments per kilobase of transcript per million mapped reads and these levels remained extremely low after *FKBP5*-silencing. DEX treatment led to 4.6-fold increase in levels; however, they remained <0.1 fragments per kilobase of transcript per million mapped (data not shown). Likewise, previous studies performed in our laboratory found no differences in FKBP52 levels, a positive regulator of GR signaling (30), in leiomyoma vs paired myometrial tissue (data not shown). Importantly, DEX is a synthetic glucocorticoid that does not require HSD11β1/β2 for activation/inactivation and interacts with GR differently than endogenous glucocorticoids (29). These considerations suggest that upregulation of HSD11β1 in our leiomyoma and myometrial cell cultures occurred via FKPB51 upregulation either directly or indirectly through an unknown third-party mediator.

Interestingly, a prior study noted differential GR binding and GR target gene regulation with mediator of RNA polymerase II transcription subunits 1 and 14 (MED1, MED14) (31). The role of a different mediator subunit, MED12, has been previously implicated in the pathogenesis of leiomyoma, with a recent meta-analysis finding MED12 mutations in 55.8% of leiomyomas analyzed. Leiomyoma development from cells with mutated MED12 is thought to occur through increased AKT signaling and decreased cyclin C-CDK8/19 kinase activity, leading to myometrial cell growth and proliferation (32). However, given that MED12 mutations disrupt mediator complex activity, altering transcriptional activity in leiomyomas (33), MED12 interaction with GR signaling, and/or FKBP51 may be interesting to investigate in future studies.

Given that the current leiomyoma therapies primarily target estrogen and progesterone signaling or block GnRH signaling, our findings suggest a potentially new approach for treating leiomyoma. Though further omics studies are needed to clarify how FKBP51-GR directly regulates intracellular HSD11β1 and alters the transcriptional activity in leiomyoma cells, we found that DEX treatment in the presence of *FKBP5* induces *HSD11B1* expression to a greater extent in leiomyoma cells compared to normal myometrium. This leads to the upregulation of extracellular matrix genes and downregulation of smooth muscles genes. Notably, this enhanced *HSD11B1* expression in leiomyoma was absent following *FKBP5* knockdown, highlighting the interconnectivity between *FKBP5* and *HSD11B1* in inducing a myofibroblast phenotype.

In conclusion, blocking FKBP51-meditated GR transcriptional activity, along with inhibiting HSD11β1 expression and/or activity, could disrupt the “FKBP51-GR-HSD11β1” cycle in leiomyomatous uteri. This disruption may reduce extracellular matrix protein production, possibly limiting leiomyoma formation and growth (Fig. 7). Our findings provide a new treatment strategy, particularly for patients who cannot use hormone therapy and/or wish to conceive.

## Data Availability

The RNA-sequencing datasets presented in this study can be found in the online NCBI GEO repository, accession number GSE292403. The repository can be accessed using the link here (20): https://www.ncbi.nlm.nih.gov/geo/query/acc.cgi?acc=GSE292403.

## Abbreviations

C-DEX: dexamethasone-treated control cells
DEG: differentially expressed gene
DEX: dexamethasone
ECM: extracellular matrix
FKBP51: FK506-binding-protein-51
FKD-DEX: dexamethasone-treated *FKBP5*-silenced cells
GR: glucocorticoid receptor
HESC: human endometrial stromal cell
HSD11β1: hydroxysteroid 11-β dehydrogenase 1
IPA: Ingenuity Pathway Analysis
PR: progesterone receptor
qPCR: quantitative polymerase chain reaction
RNA-seq: RNA-sequencing
TBS: Tris-buffered saline
